# Video game-facilitated group activity for quality of life and social function in chronic schizophrenia inpatients: A randomized controlled pilot trial

**DOI:** 10.1192/j.eurpsy.2026.12181

**Published:** 2026-07-31

**Authors:** W.-Y. Chiang, Y.-J. Shih, Y.-J. Yang

**Affiliations:** 1 Department of General Psychiatry; 2 Department of Nursing, Tsao-Tun Psychiatric Center, Nan-Tou County; 3 Department of Health Administration, College of Medical and Health Science, Asia University, Taichung City, Taiwan

## Abstract

**Introduction:**

Schizophrenia is characterized by chronicity and a pervasive impact on patients’ social functioning and quality of life. While pharmacological treatment can mitigate positive symptoms, negative symptoms, social dysfunction, and impaired quality of life require psychosocial interventions. Group-based interventions are among those known to improve social functioning and quality of life, but the benefits of video game-facilitated group activities for this population remain unclear.

**Objectives:**

To evaluate the effectiveness of video game-facilitated group activity programs on quality of life and social function in chronic schizophrenic inpatients.

**Methods:**

Schizophrenic inpatients aged 20–65 years at Tsao-Tun Psychiatric Center (TTPC) in Taiwan were recruited in this study. Participants were randomly assigned to either a 12-week intervention (three 50-minute video game group sessions weekly plus usual care) or to a control group (usual care only). Social function and quality of life was assessed using the Personal and Social Performance Scale (PSP) and the World Health Organization Quality of Life-Brief Version (WHOQOL-BREF), Taiwan version. Demographic data, physiological profiles, cognitive function, psychiatric conditions, and physical fitness were also collected for analysis. Analyses were performed with R, and statistical significance was set at the 5% level. This study was approved by the TTPC ethical review committee and registered (NCT07148609).

**Results:**

This preliminary report summarizes our first wave of participants. Twenty patients were enrolled, with their basic demographic data shown in Table 1. At the endpoint, the intervention group exhibited significant improvement in personal and social relationships, as measured by the PSP (p=0.002), and in the language subscale of the Montreal Cognitive Assessment (MoCA) (p=0.023). However, no significant difference was found in other outcome measurements between groups.

**Image 1:**
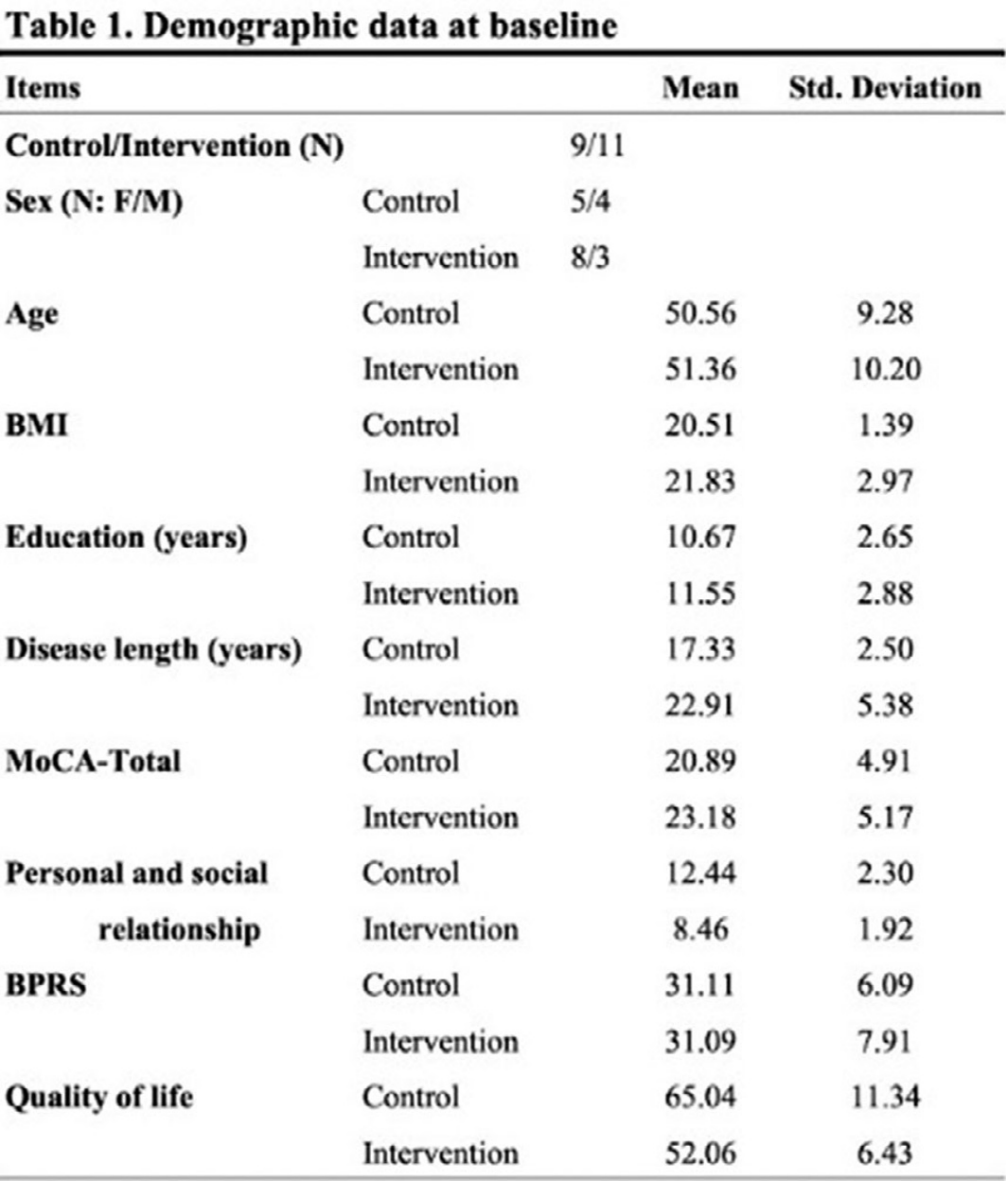
Image 1: Long description.

**Conclusions:**

Our study showed that video game-facilitated group activity intervention significantly improved personal and social relationships in chronic schizophrenic inpatients and may enhance the language performance in cognitive assessment. These results highlight the clinical implications of embedding video game programs to promote social interactions in this population and warrant further studies.

**Disclosure of Interest:**

None Declared

## Long descriptions

### Image 1: Long description

Table 1 titled Demographic data at baseline. The table has three main columns: Items, Mean, and Std. Deviation.

* Control/Intervention (N): 9/11.

* Sex (N: F/M): Control is 5/4; Intervention is 8/3.

* Age: Control Mean 50.56 (Std. Deviation 9.28); Intervention Mean 51.36 (Std. Deviation 10.20).

* B M I: Control Mean 20.51 (Std. Deviation 1.39); Intervention Mean 21.83 (Std. Deviation 2.97).

* Education (years): Control Mean 10.67 (Std. Deviation 2.65); Intervention Mean 11.55 (Std. Deviation 2.88).

* Disease length (years): Control Mean 17.33 (Std. Deviation 2.50); Intervention Mean 22.91 (Std. Deviation 5.38).

* MoCA-Total: Control Mean 20.89 (Std. Deviation 4.91); Intervention Mean 23.18 (Std. Deviation 5.17).

* Personal and social relationship: Control Mean 12.44 (Std. Deviation 2.30); Intervention Mean 8.46 (Std. Deviation 1.92).

* B P R S: Control Mean 31.11 (Std. Deviation 6.09); Intervention Mean 31.09 (Std. Deviation 7.91).

* Quality of life: Control Mean 65.04 (Std. Deviation 11.34); Intervention Mean 52.06 (Std. Deviation 6.43).

Navigate back to Image 1:.

